# Biomechanical Comparisons of Different Reconstructive Techniques for Scapholunate Dissociation: A Cadaveric Study

**DOI:** 10.3390/bioengineering10111310

**Published:** 2023-11-13

**Authors:** Il-Jung Park, Seungbum Chae, Dai-Soon Kwak, Yoon-Vin Kim, Seunghun Ha, Dohyung Lim

**Affiliations:** 1 Department of Orthopedic Surgery, College of Medicine, The Catholic University of Korea, Seoul 06591, Republic of Korea; jikocmc@catholic.ac.kr (I.-J.P.); kyb901@hanmail.net (Y.-V.K.); hsh5257@gmail.com (S.H.); 2 Department of Orthopedic Surgery, Daegu Catholic University Medical Center, School of Medicine, Daegu Catholic University, Deagu 42472, Republic of Korea; genichess@naver.com; 3 Chae Seungbum Orthopedic Clinic, Deagu 42471, Republic of Korea; 4 Catholic Institute for Applied Anatomy, Department of Anatomy, College of Medicine, The Catholic University of Korea, Seoul 06591, Republic of Korea; 5 Department of Mechanical Engineering, Sejong University, Seoul 05006, Republic of Korea; dli349@sejong.ac.kr

**Keywords:** scapholunate dissociation, reconstructive techniques, cadaveric biomechanical study

## Abstract

There are many techniques for the treatment of chronic scapholunate dissociation. The three-ligament tenodesis (3LT) is used most widely, but reconstruction of the dorsal ligament alone may not provide sufficient stability. The Mark–Henry technique (MHT) compensates for the insufficient stability of 3LT by additional reconstruction of the volar ligament, but the procedure is complex. The SwiveLock technique (SWT), a recently introduced method, provides stability by using autologous tendons with synthetic tapes, but lacks long-term clinical results. To perform biomechanical comparisons of different reconstructive techniques for scapholunate dissociation using a controlled laboratory cadaveric model. Eleven fresh-frozen upper-extremity cadaveric specimens were prepared. The scapholunate distance, scaphoid rotation, and lunate rotation of the specimens were measured during continuous flexion–extension and ulnar–radial deviation movements. The data were collected using a wrist simulator with a linear guide rail system (tendon load/motion-controlled system) and a motion capture system. Results were compared in five conditions: (1) intact, (2) scapholunate dissociation, (3) SWT, (4) 3LT, and (5) MHT. Paired *t*-test was employed to compare the biomechanical characteristics of intact wrists to those of scapholunate dissociated wrists, and to those of wrists after each of the three reconstruction methods. SWT and MHT were effective solutions for reducing the widening in scapholunate distance. According to the radioscaphoid angle, all three reconstruction techniques were effective in addressing the flexion deformity of the scaphoid. According to the radiolunate angle, only SWT was effective in addressing the extension deformity of the lunate. In terms of scapholunate angle, only the results after SWT did not differ from those of the intact wrist. The SWT technique most effectively improved distraction intensity and rotational strength for the treatment of scapholunate dissociation. Taking into account the technical complexity of 3LT and MHT, SWT may be a more efficient technique to reduce operating time and minimize complications due to multiple incisions, transosseous tunnels, and complicated shuttling.

## 1. Introduction

There is currently no consensus on the best operative treatment for chronic scapholunate dissociation with an irreparable scapholunate interosseous ligament (SLIL) but without degenerative change [1]. Numerous reconstructive surgeries have been described in previous studies. Among them, dorsal capsulodesis, bone–ligament–bone autografts, scapholunate axis method (SLAM), reduction and association of the scaphoid and lunate (RASL), and a variety of tendon reconstruction methods are currently used [2,3,4,5,6,7,8]. However, the results of these procedures are unsatisfactory and the single most effective treatment for chronic scapholunate instability is yet to be determined [9].

**Figure 1:**
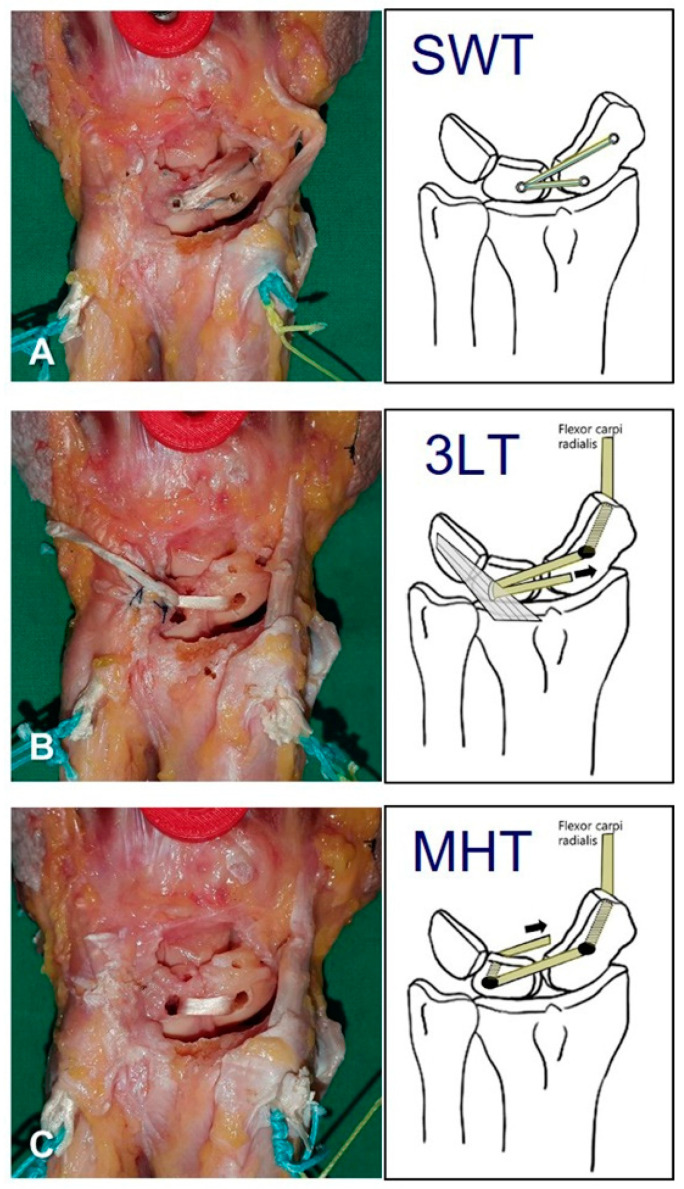
Reconstructed specimens and schematic diagrams of the three different reconstructions. (**A**) SwiveLock technique (SWT), (**B**) three-ligament tenodesis (3LT), and (**C**) Mark–Henry technique (MHT).

The purpose of reconstruction is to address the scapholunate interval widening and scaphoid flexion deformity to prevent scapholunate advanced collapse arthritis in the future [10]. The most used reconstruction technique at present is three-ligament tenodesis (3LT) [8]. This technique incorporates some features from previously published reconstruction procedures using a strip of the flexor carpi radialis (FCR) tendon. It enhances the action of three ligaments (scaphotrapeziotrapezoid, dorsal scapholunate, dorsal radiotriquetral ligament) and has therefore been named the 3LT procedure [8]. Although 3LT is a reliable technique, with favorable results reported by several authors, ligamentous loosening did result in the rapid recurrence of radiological abnormalities and frequent complications [11,12,13]. To compensate for the ligamentous loosening of 3LT, the Mark–Henry technique (MHT) was introduced [14]. This technique reconstructs both the volar and dorsal limbs of the SLIL and addresses the multidirectional nature of scapholunate instability. However, the surgical procedure was somewhat complicated, and there were complications such as fractures and avascular necrosis of the lunate [15]. Many tendon-to-ligament reconstructions for chronic ligament injuries have been shown to lead to the recurrence of radiological deformities due to the ligament loosening over several years [11,16,17]. The leading causes of this loosening were thought to be the stretching of the grafted tendon and the weakening of the fixation site [18,19,20,21]. The use of synthetic tape may mitigate the stretching and weakening of the grafted tendon, and suture anchors can enhance the fixation strength. In light of these considerations, the SwiveLock technique (SWT) using autologous tendons and synthetic tape was introduced. Autologous tendon graft and synthetic tape are secured at the center point of the lunate and the proximal pole and distal pole of the scaphoid dorsum by anchors. This procedure is straightforward, but lacks clinical and basic research results [10,22]. We performed biomechanical comparisons of different reconstructive techniques for scapholunate dissociation using a controlled laboratory cadaveric model (Figure 1). Through this experiment, we aimed to answer the following questions. First, by comparing 3LT and MHT, we tried to clarify whether volar ligament reconstruction is absolutely necessary. Second, by comparing 3LT and SWT, we ascertained that an even simpler method can achieve sufficiently effective results. The purpose of this study was to compare the biomechanical characteristics of three different reconstructive techniques for scapholunate dissociation in a cadaveric model.

## 2. Materials and Methods

This study was approved by the Institutional Cadaver Research Committee of the College of Medicine, The Catholic University of Korea (No. R21-A007). Written informed consent for use of the cadaver and consent for use in future research on the related materials were provided by all donors or their authorized representatives.

In this study, 11 arms (9 male, 2 female) were used from 9 fresh cadavers (donated to the College of Medicine, The Catholic University of Korea). The mean age was 80.1 years (range, 71–91). All specimens were macroscopically intact and did not exhibit any gross pathology. Each specimen was thawed at room temperature for 12 h before preparation. Two senior surgeons performed every surgical procedure together to reduce technical variation.

### 2.1. Specimen Preparation

Midline incisions of the forearm were made at both dorsal and volar aspects, followed by skin flap elevation. All soft tissues proximal to the hand were dissected, except for the interosseous membrane, wrist capsule, and tendons. The wrist flexor tendons (flexor carpi radialis (FCR), flexor carpi ulnaris (FCU)), wrist extensor tendons (extensor carpi radialis brevis (ECRB), extensor carpi radialis longus (ECRL), extensor carpi ulnaris (ECU)), and the deep flexor tendons of the fingers (flexor digitorum profundus (FDP; four tendons)) were left intact. Sufficient moisturization by normal saline was maintained throughout the preparation and testing process of the specimens. The neutral rotation of the forearm (with the humerus positioned vertically and the elbow flexed at 90°) was sustained by fixation with a cortical screw. Subsequent forearm transection took place 16 cm proximal to the tip of the radial styloid. The proximal end of the specimen was potted in the cylindrical mold with resin (Z-Grip, Evercoat, Cincinnati, OH, USA) to mount a wrist simulator. During the potting procedure, a specialized jig and stand were used to fix the mold, preserving the original anatomical axis. Extreme caution was needed to ensure that a neutral alignment was maintained in both the sagittal and coronal planes. Locking and running Krackow stitches using 2-0 braided sutures were placed in each individual tendon and connected to strings to allow for subsequent weight loading. The ECRB and ECRL tendons were sutured together, and the four FDP tendons were sutured side-to-side for equal load transfer. After preparation of the specimens, the specimen was securely mounted in original axial alignment on a custom wrist simulator.

### 2.2. Biomechanical Testing

**Figure 2:**
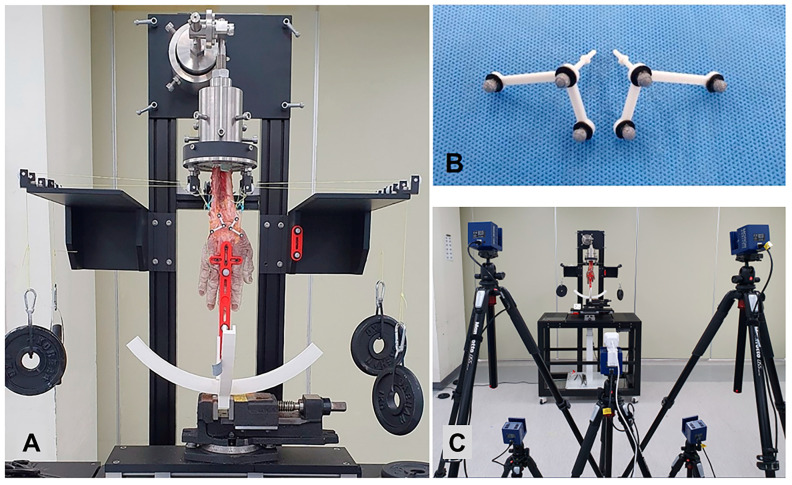
The specimen fixed to the wrist simulator. (**A**) The simulator was designed to be able to load the muscle force. A custom-designed orthogonal marker set was attached to the dorsal aspect of the scaphoid and lunate. (**B**) The orthogonal marker set consisted of three optical markers and a column inserted into the bone at a constant depth. (**C**) The motion cameras’ configurations.

The prepared specimen was fixed to the wrist simulator with the proximal end of the specimen attached to the cylinder mold, facing up. The simulator was designed to be able to load the muscle force (Figure 2A). For muscle loading, we prepared weight suspensions. The strings attached to the Krackow stitches of each individual tendon were positioned through guide rods to achieve physiologic lines of pull. Small metal hooks were used for weight suspension on the prepared tendons. A 10 N weight was applied to the FCU, FCR, ECU and the combined ECRL/ECRB tendons. A 20 N weight was applied to the four combined FDP tendons to create a clenched fist position. The magnitudes of these loads are not exactly the same as those used by Pollock et al. [1], as we observed that different weights were required to produce the appropriate wrist motion.

To measure the movement of the scaphoid and lunate, a custom-designed orthogonal marker set was attached to the dorsal aspect of each carpal bone. The orthogonal marker set consisted of three optical markers and a column inserted into the bone at constant depths (Figure 2B). To measure the flexion–extension angle of the wrist joint, two optical markers were attached on the dorsal aspect of the 3rd metacarpus and another two optical markers were placed on the vertical axis of the simulator.

We used five motion analysis cameras (Kestral 1300, MotionAnalysis, Rohnert Park, CA, USA) to measure marker positions (Figure 2C). To check the measurement accuracy of the five motion analysis cameras, a 200 mm length scale was measured repeatedly. Measurement results of 199.98–200.03 mm were confirmed, and the measurement accuracy was about 1/10 mm. A guide rail made by a 3D printer was installed and used to reproduce the constant movement path of the wrist joint extension–flexion and ulnar–radial deviation. The condition of each cadaveric specimen was recorded in the following order: intact, scapholunate dissociation, SWT, 3LT, and MHT. We implemented passive movements under muscle loading in the order of extension–flexion and ulnar–radial deviation on the intact specimen. The movements of each marker were recorded at 20 Hz.

After recording the results of the intact wrist, scapholunate dissociation was surgically inflicted on each wrist. The SLIL is the primary stabilizer, and the radioscaphocapitate (RSC), scaphotrapeziotrapezoid (STT), and dorsal intercarpal (DIC) ligaments are the main secondary stabilizers of the scapholunate articulation [23,24,25]. By using a scalpel, all three (dorsal, membranous, and palmar) portions of the SLIL were dissected, followed by division of the RSC, STT and DIC ligaments. After ligament sectioning, the scapholunate complex was grossly unstable. We implemented passive movements under muscle loading in the order of extension–flexion and ulnar–radial deviation, and the movements of each marker were recorded at 20 Hz.

After recording the results of scapholunate dissociation, surgical reconstructions were made in the order of SWT, 3LT, and MHT. First, reconstruction using SWT was performed. Drill holes for the autologous palmaris longus tendon and synthetic tapes (SutureTape; Arthrex, Naples, FL, USA) were created at the lunate center, the scaphoid proximal pole dorsum, and scaphoid distal pole dorsum. Hole placement is crucial, especially at the proximal pole of the scaphoid, as breakage of the cortex will lead to an insecure graft fixation. Suture anchors (3.5-mm DX SwiveLock SL; Arthrex) were used for SWT reconstructions, and the anchors were inserted at 90 degrees, as anchor insertion at obtuse angles may reduce the pullout strength.

After recording the results of SWT, the suture anchors were removed, and a 3LT reconstruction was performed. A half-slip of the FCR tendon was divided from the tendon and proximally cut loose, and the other half-slip remained distally attached. The loosened half-slip of the tendon was then threaded through a 3.2 mm volar-to-dorsal tunnel in the scaphoid using a wire tendon shuttle. After being pulled tightly across the lunate trough to emerge through a slit in the dorsal radiotriquetral ligament, it was taken back across the lunate, where it was finally woven into the tendon slip with a 3-0 suture.

After recording the results of 3LT, the sutures of 3LT reconstruction were released to free the FCR tendon half-slip, and MHT reconstruction was performed. A lunate tunnel was made, and the loose FCR tendon half-slip was drawn through the tunnel from the dorsal side to the volar side. The tendon half-slip was put into tension as it emerged on the radial side of the remaining FCR tendon and woven into the remaining FCR tendon and volar capsule with a 3-0 suture. The results were recorded under the same circumstances as in the intact condition, scapholunate dissociation condition, and after each different reconstruction.

### 2.3. Statistical Analysis

Previously obtained pilot study data were used for sample size calculation. A minimum sample size of 10 specimens was considered adequate to obtain an α of 0.05 and power of 0.8 to demonstrate statistically significant difference. The continuous motion data of the scaphoid and lunate during wrist flexion–extension ulnar–radial deviation were analyzed using the scientific programming language Matlab (R2021, Mathworks, Natick, MA, USA). The distance between the scaphoid and lunate, the angle between scaphoid and radius, the angle between lunate and radius, and the angle between scaphoid and lunate were analyzed. After confirming the normal distribution by performing the Shapiro–Wilk test at each position, paired *t*-test was employed to compare the biomechanical characteristics of intact wrists to that of scapholunate dissociated wrists, and to that of wrists after each of the three reconstruction methods. *p* < 0.05 was considered to be statistically significant.

## 3. Results

Complete SLIL sectioning followed by the division of the RSC, STT and DIC ligaments resulted in a typical pattern of scapholunate dissociation. After scapholunate dissociation, all four parameters worsened. After the three different reconstructions, all four parameters improved in almost every wrist position. All measurement results are provided as Supplementary Materials (Table S1).

### 3.1. Scapholunate Distance

**Figure 3:**
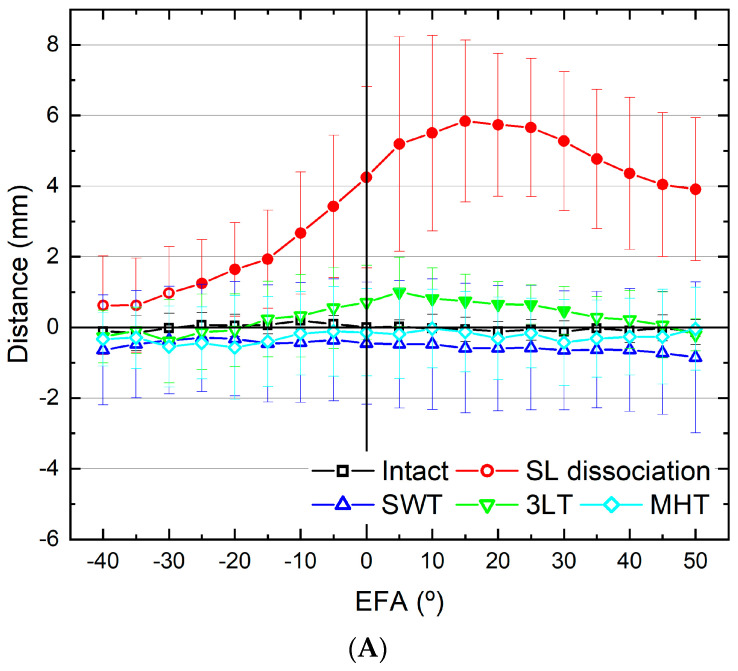

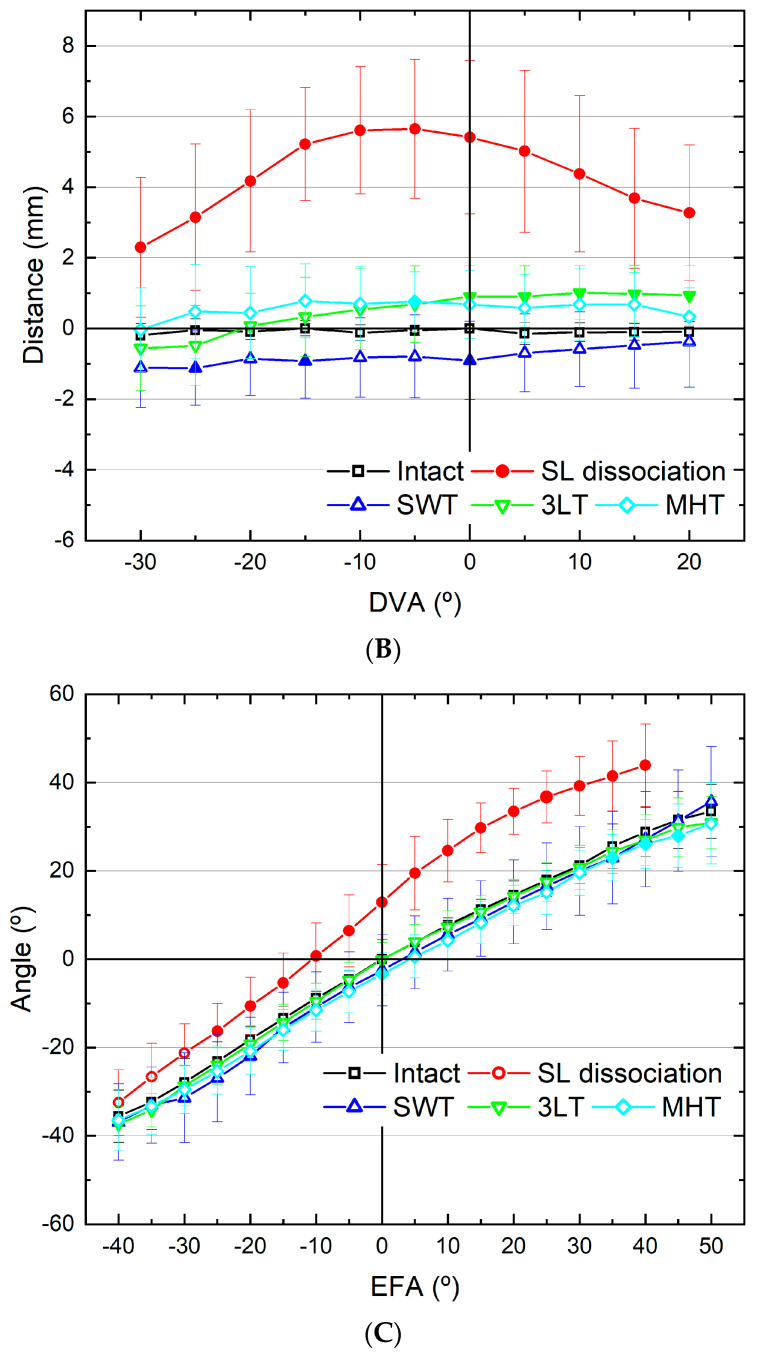

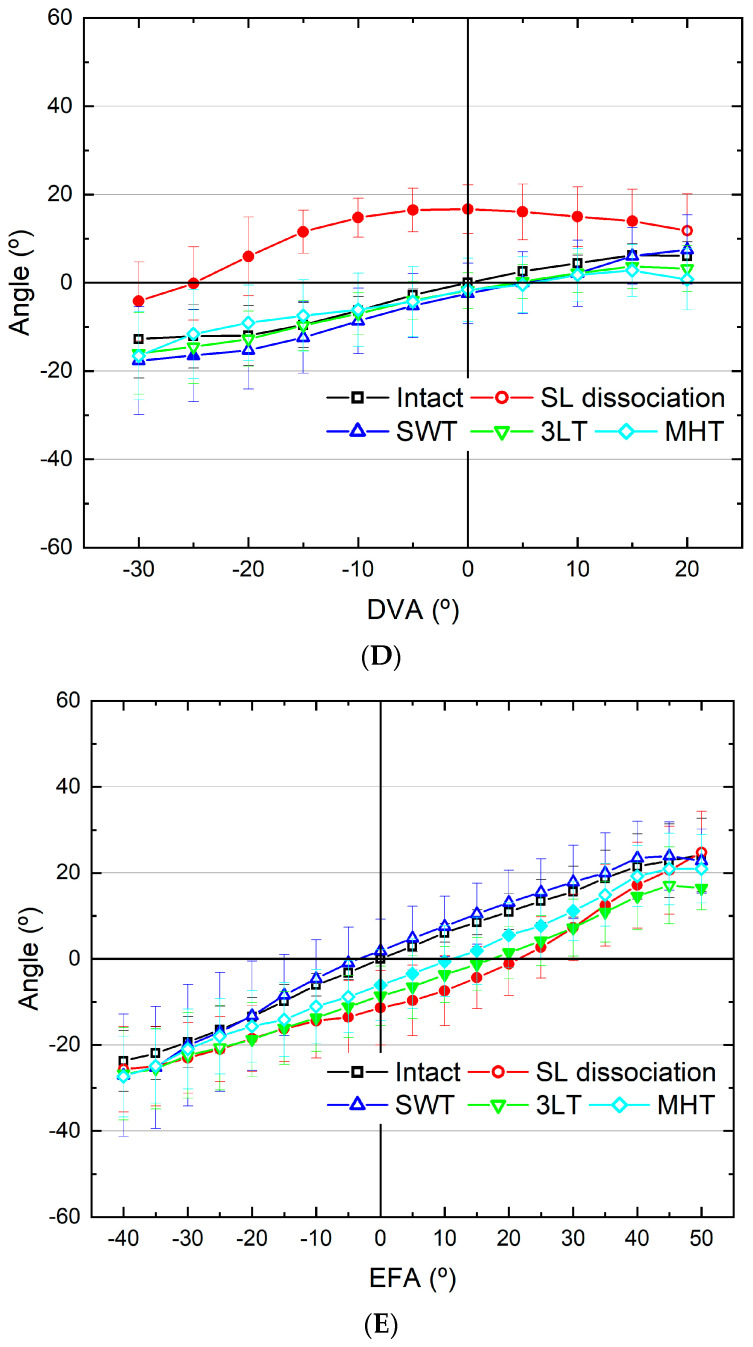

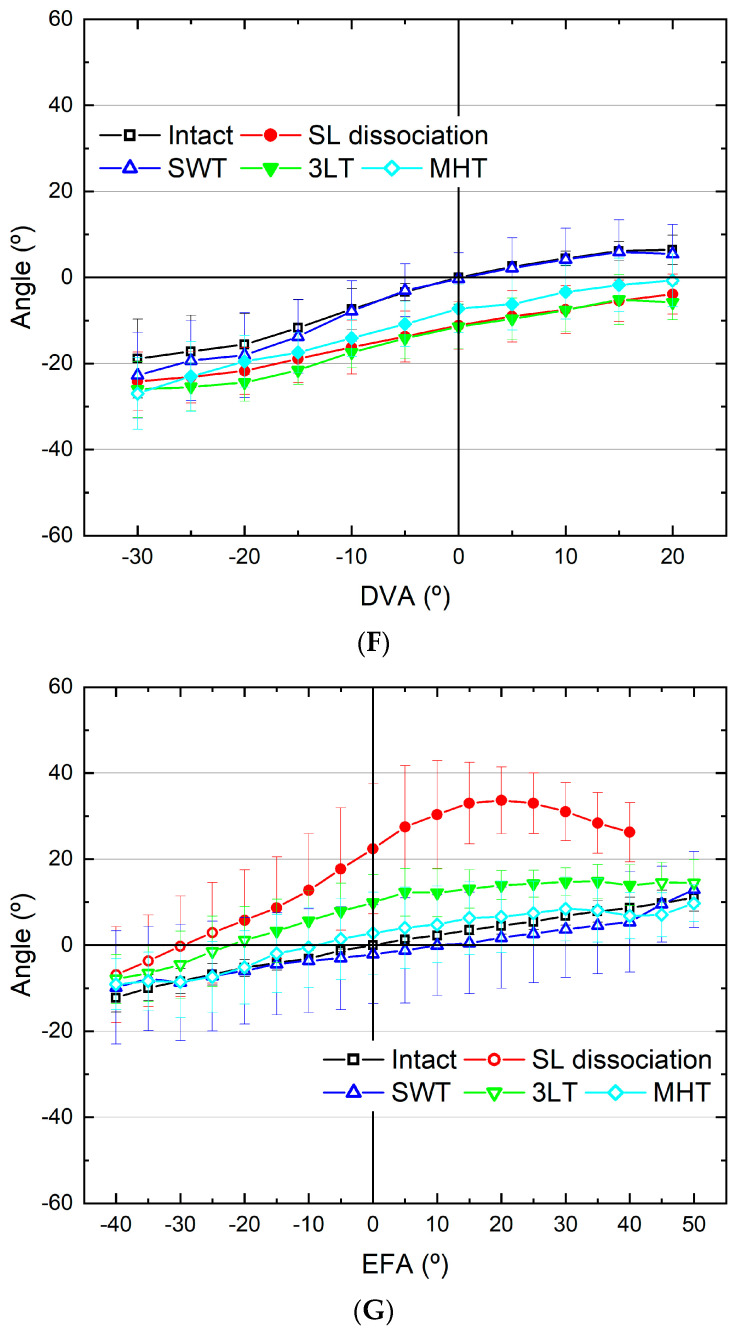

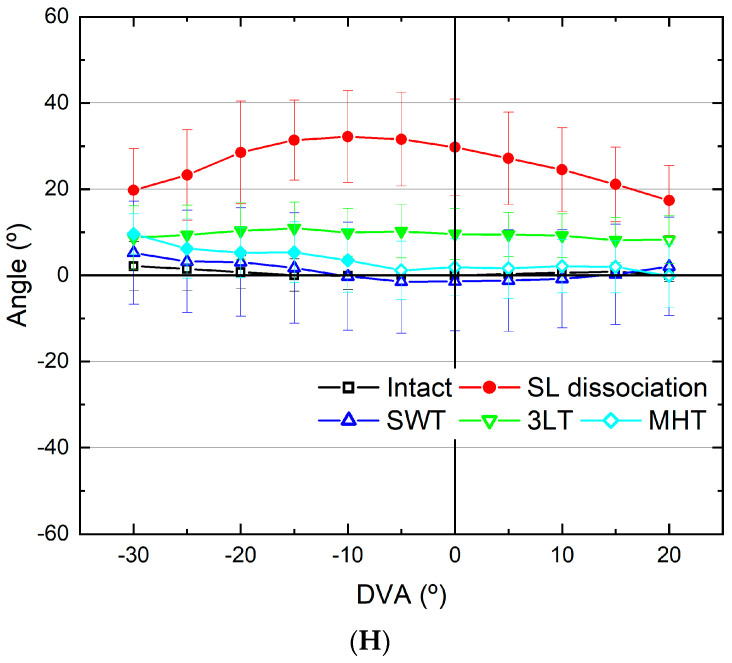
Results from continuous motion data of scaphoid and lunate during wrist extension –flexion and ulnar–radial deviation. The filled shapes (circle, triangle, inverted triangle and rhombus) showed statistically significant values (*p* < 0.05) compared to the intact wrists. SL dissociation = scapholunate dissociation; SWT = SwiveLock technique, 3LT = three-ligament tenodesis, MHT = Mark–Henry technique, EFA = extension to flexion angle, DVA = ulnar to radial deviation angle. (**A**,**B**) The scapholunate distance after SWT and MHT was not different from that of the intact wrists, but 3LT had wider scapholunate distance than the intact wrists. (**C**,**D**) The radioscaphoid angle after 3LT, SWT and MHT did not differ from that of the intact wrists. (**E**,**F**) The radiolunate angle after SWT did not differ from that of the intact wrists, but 3LT and MHT had larger radiolunate angles than the intact wrists. (**G**,**H**) The scapholunate angle after SWT did not differ from that of the intact wrists, but 3LT and MHT had larger scapholunate angles than the intact wrists. In some SL dissociation samples, irregular and rapid movement (pop-up) of the scaphoid bone was observed at high flexion angles (45 and 50 degrees) in (**C**,**G**).

The scapholunate distance significantly increased after scapholunate dissociation (Figure 3A,B, Tables S1 and S2). The widest gap was observed at 15° of flexion and 10° of ulnar deviation. The scapholunate distance was reduced after all three reconstructions. There were statistically significant differences between 3LT and intact wrists at 5°, 10°, 15°, 20°, 25°, and 30° flexion (*p* = 0.03, 0.04, 0.02, 0.01, 0.01 and 0.04, respectively) and 0°, 5°, 10°, 15°, and 20° radial deviation (*p* = 0.01, 0.01, 0.00, 0.01 and 0.01, respectively). There was no statistically significant difference between MHT and intact wrists except for 5° ulnar deviation (*p* = 0.04). There was no statistically significant difference between SWT and intact wrists except for 25°, 15°, and 0° ulnar deviation (*p* = 0.03, 0.04 and 0.04, respectively). Interestingly, the scapholunate distances after SWT were mostly narrower than those of the intact wrists, although the difference was not statistically significant. This was thought to be the effect of over-tightening. In summary, the scapholunate distance after SWT and MHT did not differ from that of the intact wrists. However, 3LT had a wider scapholunate distance than the intact wrists.

### 3.2. Radioscaphoid Angle

The radioscaphoid angle significantly increased after scapholunate dissociation and improved after the three reconstructions in every wrist position (Figure 3C,D, Tables S3 and S4). There was no statistically significant difference between MHT and the intact wrists, except for 0°, 35°, 40° and 45° flexion (*p* = 0.04, 0.04, 0.04 and 0.02, respectively). Both 3LT and SWT have no statistically significant difference compared to the intact wrists at every wrist position. In summary, the radioscaphoid angle after 3LT, SWT and MHT did not differ from that of the intact wrists.

### 3.3. Radiolunate Angle

The radiolunate angle significantly increased after scapholunate dissociation (Figure 3E,F, Tables S5 and S6). There were statistically significant differences between 3LT and intact wrists at almost every wrist position, except for 40°, 35°, 30° and 25° extension (*p* = 0.23, 0.16, 0.20 and 0.10, respectively) and 45° flexion (*p* = 0.07). There were statistically significant differences between MHT and intact wrists at 0°, 5°, 10°, 15°, 20°, 25° and 30° flexion (*p* = 0.03, 0.03, 0.02, 0.02, 0.04, 0.02 and 0.04 respectively) and almost all ulnar and radial deviations. There was no statistically significant difference between SWT and intact wrists in every wrist position. In summary, the radiolunate angle after SWT did not differ from that of the intact wrists. However, 3LT and MHT had larger radiolunate angles than the intact wrists.

### 3.4. Scapholunate Angle

The scapholunate angle significantly increased after scapholunate dissociation (Figure 3G,H, Tables S7 and S8). There were statistically significant differences between 3LT and intact wrists in almost every wrist position, except for 40°, 35°, 30°, 25° and 20° extension (*p* = 0.29, 0.26, 0.29, 0.12 and 0.05, respectively) and 45°, 50° flexion (*p* = 0.06, 0.49, respectively). There were statistically significant differences between MHT and intact wrists at 30°, 25°, 20°, 15° and 10° ulnar deviation (*p* = 0.00, 0.00, 0.02, 0.01 and 0.01, respectively). There was no statistically significant difference between SWT and intact wrists in any wrist position. In summary, the scapholunate angle after SWT did not differ from that of the intact wrists. However, MHT had a larger scapholunate angle than the intact wrists at ulnar deviation. 3LT had larger radiolunate angles than the intact wrists in every wrist position.

## 4. Discussion

The purpose of this study was to compare the biomechanical characteristics of three different reconstructive techniques for scapholunate dissociation by using a cadaveric model. Among the three techniques, the SWT technique most effectively improved distraction intensity and rotational strength for the treatment of scapholunate dissociation. Garcia-Elias and colleagues evaluated SLIL injuries and proposed a classification system with six stages. The Stage 3 and 4 SLIL injuries are characterized by a complete, non-repairable ligament, with either a normally aligned scaphoid or reducible deformity [8]. In these circumstances, SLIL reconstruction is considered using various types of previously described reconstructive procedures. The reconstructive procedure aims to address the scapholunate interval widening, scaphoid flexion deformity, and extension deformity of the lunate [10]. The scapholunate distance after SWT and MHT was not different from that of the intact wrists, but 3LT had a wider scapholunate distance than the intact wrists. Thus, SWT and MHT were effective in addressing the scapholunate distance widening. The radioscaphoid angle after 3LT, SWT and MHT did not differ from that of the intact wrists. Therefore, all three reconstruction techniques are effective in addressing the flexion deformity of the scaphoid. The radiolunate angle after SWT did not differ from that of the intact wrists, but 3LT and MHT had larger radiolunate angles than the intact wrists. As a result, only SWT was effective in addressing the extension deformity of the lunate. The scapholunate angle after SWT did not differ from that of the intact wrists, but 3LT and MHT had larger scapholunate angles than the intact wrists. Based on these findings, SWT was most effective in improving distraction intensity and rotational strength for the treatment of scapholunate dissociation.

Some of the reasons for reconstruction failures in the past were the stretching out of the tendon graft and weak fixation [17,18,19,20]. SWT is a reconstructive technique using the autologous tendon with synthetic tape augmentation. It requires two limbs: a short-transverse limb that corrects the scapholunate interval (coronal plane correction) and a long-oblique limb that corrects scaphoid rotary subluxation and dorsal intercalated segment instability (sagittal plane correction). The purpose of the synthetic tape augmentation is reinforcement of the biologic reconstruction with sutures to provide immediate biomechanical strength and support before graft incorporation [26,27,28]. The anchors in the scaphoid and lunate are firmly wedged into the bone tunnel with interference securing tight fixation. Even though clinical results for this procedure are limited, it is considered to overcome the disadvantages of various other reconstruction methods.

The dorsal SLIL is known to be substantially thicker and stronger than the volar SLIL. It plays an important role in resisting scaphoid pronation and flexion and proximal pole dorsal subluxation relative to the lunate [5]. Therefore, reconstruction of the dorsal SLIL has been the main focus for a prolonged period of time [21]. However, sole reconstruction of the dorsal ligament may result in over-tightening on the dorsal side, along with a hinge effect with gapping on the volar side [29,30]. In the present study, the scapholunate distance after SWT was mostly narrower than that of the intact wrists, although the difference was not statistically significant. However, all tendon-to-ligament reconstructions have the tendency of early creeping and delayed elongation. The clinical effects of over-tightening have not been elucidated. In any case, the surgeon should try to avoid the stiffness caused by excessive tightening.

Various measurement methods have been used for biomechanical wrist experiments. The plane radiograph technique is common [1,29,30,31,32,33,34]. Since this method measures an image projected on a two-dimensional plane, it cannot be free from errors caused by alignment differences during image-taking and cannot be free from inter- and intra-reliability. To solve these shortcomings, some researchers have used a 3D digitizer [10,15,35,36]. The digitizer is a method of measuring the three-dimensional coordinates of specific points, and the position change in the metacarpal bones can be measured in a specific posture. The advantage is that errors due to two-dimensional measurements using radiographs can be completely prevented. However, errors may still occur while using digitizers during the repetitive pointing at specific coordinates. Continuous movement cannot be measured, and the disadvantage is that the wrist needs to be set in a specific posture for measurement. The optical marker and motion analysis device applied in this study can continuously measure the flexion–extension and deviated motion processes during movement. Moreover, since the observer does not intervene in the measurement after initiation of the experiment, more reproducible and precise results than the previous methods can be obtained.

The present study has its limitations. First, this was a time-zero cadaveric biomechanical study, which cannot reflect the actual in vivo clinical situation. The clinical postoperative strength and stiffness after the reconstruction procedures are unknown. Also, although there are no reported adverse human reactions to the synthetic material, the long-term effects of the synthetic tape could not be studied. Second, in terms of the reconstruction technique sequence, the ideal reconstruction order was SWT, 3LT, and MHT. The predetermined reconstruction sequence may have effects on the strength of reconstruction. However, as the overall strength of 3LT is lower than that of MHT, the effect of the reconstruction sequence on reconstruction strengths may be considered insignificant. Finally, carpal bones have complex interrelationships that depend on wrist position and the direction of wrist movement. The four parameters (scapholunate interval, radioscaphoid angle, radiolunate angle, and scapholunate angle) alone may not comprehensively demonstrate the exact static and dynamic relationships of the carpal bones. However, there is no known parameter that better reflects the relationships of the carpal bones than the four variables, which is why they are widely used in many other experiments related to scapholunate dissociation.

Despite these limitations, this study provides important information about the biomechanical characteristics of the three different reconstructive techniques for the treatment of SLIL injury and may be useful in the management of scapholunate dissociation. Future studies, such as clinical series using the SWT in cases of dynamic and static scapholunate dissociation, should be considered.

Considering the technical complexity of 3LT and MHT, SWT may be a more efficient technique to reduce operating time and minimize complications due to multiple incisions, transosseous tunnels, and complicated shuttling. The over-tightening effect may occur on the dorsal side after SWT, so surgeons should be alert and try to avoid stiffness caused by excessive tightening.

## Data Availability

All data are presented in the article. Instrumental readings are available upon request from the corresponding author.

## Supplementary Materials

The following supporting information can be downloaded at: https://www.mdpi.com/article/10.3390/bioengineering10111310/s1, Tables S1–S8: Mesaurement results.

## References

[1] Pollock P.J., Sieg R.N., Baechler M.F., Scher D., Zimmerman N.B., Dubin N.H. (2010). Radiographic evaluation of the modified Brunelli technique versus the Blatt capsulodesis for scapholunate dissociation in a cadaver model. J. Hand Surg..

[2] Blatt G. (1987). Capsulodesis in reconstructive hand surgery. Dorsal capsulodesis for the unstable scaphoid and volar capsulodesis following excision of the distal ulna. Hand Clin..

[3] Szabo R.M. (2008). Scapholunate ligament repair with capsulodesis reinforcement. J. Hand Surg..

[4] Rosenwasser M.P., Paul S.B., Froimson A.I. (1989). Arthroplasty of the hand and wrist. Hand Clin..

[5] Yao J., Zlotolow D.A., Lee S.K. (2016). ScaphoLunate Axis Method. J. Wrist Surg..

[6] Harvey E.J., Berger R.A., Osterman A.L., Fernandez D.L., Weiss A.P. (2007). Bone-tissue-bone repairs for scapholunate dissociation. J. Hand Surg..

[7] Brunelli G.A., Brunelli G.R. (1996). A new surgical technique for carpal instability with scapholunate dissociation. Surg. Technol. Int..

[8] Garcia-Elias M., Lluch A.L., Stanley J.K. (2006). Three-ligament tenodesis for the treatment of scapholunate dissociation: Indications and surgical technique. J. Hand Surg..

[9] Imada A.O., Eldredge J., Wells L., Moneim M.S. (2022). Review of surgical treatment for chronic scapholunate ligament reconstruction: A long-term study. Eur. J. Orthop. Surg. Traumatol..

[10] Park I.J., Lim D., Maniglio M., Shin S.S., Chae S., Truong V., McGarry M.H., Lee T.Q. (2021). Comparison of Three Different Internal Brace Augmentation Techniques for Scapholunate Dissociation: A Cadaveric Biomechanical Study. J. Clin. Med..

[11] Goeminne S., Borgers A., van Beek N., De Smet L., Degreef I. (2021). Long-term follow-up of the three-ligament tenodesis for scapholunate ligament lesions: 9-year results. Hand Surg. Rehabil..

[12] Pauchard N., Dederichs A., Segret J., Barbary S., Dap F., Dautel G. (2013). The role of three-ligament tenodesis in the treatment of chronic scapholunate instability. J. Hand Surg. Eur. Vol..

[13] Wagner J.M., Stammler A., Harenberg P., Reinkemeier F., Lehnhardt M., Behr B. (2022). Did implementation of three ligament tenodesis improve patient outcome after chronic scapholunate instability? A retrospective study. Arch. Orthop. Trauma. Surg..

[14] Henry M. (2013). Reconstruction of both volar and dorsal limbs of the scapholunate interosseous ligament. J. Hand Surg..

[15] Chae S., Nam J., Park I.J., Shin S.S., McGarry M.H., Lee T.Q. (2021). Kinematic analysis of two scapholunate ligament reconstruction techniques. J. Orthop. Surg..

[16] Bain G.I., Watts A.C., McLean J., Lee Y.C., Eng K. (2013). Cable-augmented, quad ligament tenodesis scapholunate reconstruction: Rationale, surgical technique, and preliminary results. Tech. Hand Up. Extrem. Surg..

[17] Nienstedt F. (2013). Treatment of static scapholunate instability with modified Brunelli tenodesis: Results over 10 years. J. Hand Surg..

[18] Elgammal A., Lukas B. (2016). Mid-term results of ligament tenodesis in treatment of scapholunate dissociation: A retrospective study of 20 patients. J. Hand Surg. Eur. Vol..

[19] Ellanti P., Sisodia G., Al-Ajami A., Ellanti P., Harrington P. (2014). The modified Brunelli procedure for scapholunate instability: A single centre study. Hand Surg. Int. J. Devoted Hand Up. Limb Surg. Relat. Res. J. Asia-Pac. Fed. Soc. Surg. Hand.

[20] Moran S.L., Ford K.S., Wulf C.A., Cooney W.P. (2006). Outcomes of dorsal capsulodesis and tenodesis for treatment of scapholunate instability. J. Hand Surg..

[21] Nikolopoulos F.V., Apergis E.P., Poulilios A.D., Papagelopoulos P.J., Zoubos A.V., Kefalas V.A. (2011). Biomechanical properties of the scapholunate ligament and the importance of its portions in the capitate intrusion injury. Clin. Biomech..

[22] Mullikin I., Srinivasan R.C., Bagg M. (2020). Current techniques in scapholunate ligament reconstruction. Orthop. Clin. N. Am..

[23] Short W.H., Werner F.W., Green J.K., Sutton L.G., Brutus J.P. (2007). Biomechanical evaluation of the ligamentous stabilizers of the scaphoid and lunate: Part III. J. Hand Surg..

[24] Pérez A.J., Jethanandani R.G., Vutescu E.S., Meyers K.N., Lee S.K., Wolfe S.W. (2019). Role of Ligament Stabilizers of the Proximal Carpal Row in Preventing Dorsal Intercalated Segment Instability: A Cadaveric Study. J. Bone Jt. Surg. Am..

[25] Burnier M., Jethanandani R., Pérez A., Meyers K., Lee S., Wolfe S.W. (2021). Comparative Analysis of 3 Techniques of Scapholunate Reconstruction for Dorsal Intercalated Segment Instability. J. Hand Surg..

[26] De Giacomo A.F., Shin S.S. (2017). Repair of the thumb ulnar collateral ligament with suture tape augmentation. Tech. Hand Up. Extrem. Surg..

[27] Kakar S., Greene R.M., Denbeigh J., Van Wijnen A. (2019). Scapholunate ligament internal brace 360 tenodesis (SLITT) procedure: A biomechanical study. J. Wrist Surg..

[28] Park I.J., Maniglio M., Shin S.S., Lim D., McGarry M.H., Lee T.Q. (2020). Internal Bracing Augmentation for Scapholunate Interosseous Ligament Repair: A Cadaveric Biomechanical Study. J. Hand Surg..

[29] Hsu J.W., Kollitz K.M., Jegapragasan M., Huang J.I. (2014). Radiographic evaluation of the modified Brunelli technique versus a scapholunotriquetral transosseous tenodesis technique for scapholunate dissociation. J. Hand Surg..

[30] Lee S.K., Zlotolow D.A., Sapienza A., Karia R., Yao J. (2014). Biomechanical comparison of 3 methods of scapholunate ligament reconstruction. J. Hand Surg..

[31] Kang L., Dy C.J., Wei M.T., Hearns K.A., Carlson M.G. (2018). Cadaveric testing of a novel scapholunate ligament reconstruction. J. Wrist Surg..

[32] Athlani L., Pauchard N., Dautel G. (2018). Radiological evaluation of scapholunate intercarpal ligamentoplasty for chronic scapholunate dissociation in cadavers. J. Hand Surg. Eur. Vol..

[33] Slater R.R., Szabo R.M., Bay B.K., Laubach J. (1999). Dorsal intercarpal ligament capsulodesis for scapholunate dissociation: Biomechanical analysis in a cadaver model. J. Hand Surg..

[34] Howlett J.P., Pfaeffle H.J., Waitayawinyu T., Trumble T.E. (2008). Distal tunnel placement improves scaphoid flexion with the Brunelli tenodesis procedure for scapholunate dissociation. J. Hand Surg..

[35] Sladek V., Galeta P., Sosna D. (2012). Measuring human remains in the field: Grid technique, total station, or MicroScribe?. Forensic Sci. Int..

[36] Chae S., Nam J., Park I.-J., Shin S.S., McGarry M.H., Lee T.Q. (2022). Biomechanical Analysis of Three Different Reconstruction Techniques for Scapholunate Instability: A Cadaveric Study. Clin. Orthop. Surg..

